# Patients with Ankylosing Spondylitis and Low Disease Activity because of Anti-TNF-Alpha Therapy Have Higher TRAIL Levels Than Controls: A Potential Compensatory Effect

**DOI:** 10.1155/2014/798060

**Published:** 2014-05-21

**Authors:** Fernanda Genre, Raquel López-Mejías, Javier Rueda-Gotor, José A. Miranda-Filloy, Begoña Ubilla, Beatriz Carnero-López, Natalia Palmou-Fontana, Inés Gómez-Acebo, Ricardo Blanco, Trinitario Pina, Rodrigo Ochoa, Carlos González-Juanatey, Javier Llorca, Miguel A. González-Gay

**Affiliations:** ^1^Epidemiology, Genetics and Atherosclerosis Research Group on Systemic Inflammatory Diseases, Rheumatology Division, IDIVAL, 39011 Santander, Spain; ^2^Rheumatology Division, Hospital Lucus Augusti, 27003 Lugo, Spain; ^3^Oncology Division, Hospital Del Bierzo, Ponferrada, 24411 León, Spain; ^4^Rheumatology Division, Hospital General de Almansa, 02640 Albacete, Spain; ^5^Department of Epidemiology and Computational Biology, School of Medicine, University of Cantabria, IDIVAL, and CIBER Epidemiología y Salud Pública (CIBERESP), 39011 Santander, Spain; ^6^Cardiology Division, Hospital Lucus Augusti, 27003 Lugo, Spain

## Abstract

*Objective*. TRAIL is a potential biomarker of cardiovascular (CV) disease. Ankylosing spondylitis (AS) is a chronic inflammatory disease associated with metabolic syndrome (MeS) and accelerated atherosclerosis. We assessed whether disease activity, systemic inflammation, and MeS features were associated with circulating TRAIL levels in AS patients undergoing TNF-**α** antagonist infliximab therapy and if infliximab infusion modified TRAIL levels. *Methods*. We measured TRAIL serum levels in 30 nondiabetic AS patients without CV disease undergoing anti-TNF-**α** therapy, immediately before and after an infliximab infusion, and in 48 matched controls. Correlations of TRAIL levels with disease activity, systemic inflammation and MeS features, adipokines, and biomarkers of endothelial activation were evaluated. Changes in TRAIL levels following anti-TNF-**α** infusion were analyzed. *Results*. TRAIL levels were higher in AS patients than controls. TRAIL levels displayed an inverse correlation with total and LDL cholesterol. We observed an inverse correlation with QUICKI and a marginal association with HOMA-IR. We also found an inverse correlation with resistin and a marginal association with apelin and OPN. Anti-TNF-**α** infusion did not change TRAIL levels after 120′. *Conclusion*. Elevated TRAIL levels in AS patients may be the result of a compensatory mechanism to reduce CV risk in these patients.

## 1. Introduction

Ankylosing spondylitis (AS) is a chronic inflammatory disease associated with high incidence of cardiovascular (CV) mortality due to accelerated atherosclerosis [1]. In addition, AS patients often display metabolic syndrome (MeS) features, which include obesity, dyslipidemia, hypertension, alterations in glucose metabolism, including insulin resistance (IR), and also a dysregulation of adipokines [2]. Anti-TNF-*α* therapy has proven to be effective for the treatment of AS [3]. Interestingly, a single infusion of anti-TNF-*α* monoclonal antibody infliximab improved insulin sensitivity in nondiabetic AS patients [4]. Additionally, beneficial effects of anti-TNF-*α* on adipokines, biomarkers of atherosclerosis, and MeS-related biomarkers were also found in AS [5–12]. Since TNF-*α* blockade may account for biological changes that may slow the progression of atherosclerosis in AS patients [13], the analysis of the potential influence of treatment with anti-TNF-*α* drugs on disease activity, systemic inflammation, MeS, and new potential CV risk biomarkers might help us to understand the effect of these biologic agents on the mechanism associated with atherosclerosis in AS patients.

TNF-related apoptosis-inducing ligand (TRAIL) belongs to the TNF superfamily and was proposed as a potential biomarker of CV disease [14]. This protein can be expressed on the cell surface, as a transmembrane protein, or secreted as a soluble protein [15]. Apart from being involved in the apoptotic process, TRAIL also exerts anti-inflammatory and antiatherosclerotic functions [16–18].

Taking all these considerations into account, in the present study, we aimed to study if TRAIL serum levels were altered in AS patients undergoing infliximab therapy when compared to controls. We also aimed to assess potential associations between disease activity, systemic inflammation, adipokines, biomarkers of endothelial activation, and MeS features with circulating TRAIL levels in these patients. Finally, we also aimed to determine whether an anti-TNF-*α* monoclonal antibody infliximab infusion modified TRAIL levels.

## 2. Patients and Methods

### 2.1. Patients

We assessed a series of 30 patients with AS attending hospital outpatient clinics seen over 14 months (January 2009 to March 2010), who fulfilled the modified New York diagnostic criteria for AS [19]. They were treated by the same group of rheumatologists and were recruited from the Hospital Lucus Augusti (Xeral-Calde), Lugo, Spain. For the comparative analysis with AS patients, we used 48 controls matched by age, sex, ethnicity, and traditional CV risk factors, who did not have history of CV events.

For ethical reasons, patients included in the present study were not randomized to a placebo group. The same procedure has been found acceptable and followed in studies on the short-term effect of infliximab therapy on adipokines and biomarkers of endothelial cell activation in patients with rheumatoid arthritis (RA) [20–22].

Patients on treatment with infliximab seen during the period of recruitment with diabetes mellitus or with plasma glucose levels greater than 110 mg/dL were excluded. None of the patients included in the study had hyperthyroidism or renal insufficiency. Also, patients seen during the recruitment period who had experienced CV events, including ischemic heart disease, heart failure, cerebrovascular accidents, or peripheral arterial disease, were excluded. Patients were diagnosed as having hypertension if blood pressure was ≥140/90 mmHg or they were taking antihypertensive agents. Patients were considered to have dyslipidemia if they had hypercholesterolemia and/or hypertriglyceridemia (defined as diagnosis of hypercholesterolemia or hypertriglyceridemia by the patients' family physicians, or total cholesterol and/or triglyceride levels in fasting plasma were >220 mg/dL and >150 mg/dL, resp.). Obesity was defined if body mass index (BMI) (calculated as weight in kilograms divided by height in squared meters) was greater than 30.

In all cases, treatment with the anti-TNF-*α* monoclonal antibody infliximab was started because of active disease. All patients included in the current study had begun treatment with NSAIDs immediately after the disease diagnosis. All of them were still being treated with these drugs at the time of the study. At the time of this study, most patients were on treatment with naproxen 500–1000 mg/d. Although the 2010 updated recommendations facilitate initiation of TNF-*α* blockers in AS and only ask for 2 NSAIDs with a minimum total treatment period of 4 weeks [23], for the initiation of anti-TNF-*α* therapy in these series of patients recruited between January 2009 and March 2010, they had to be treated with at least 3 NSAIDs prior to the onset of infliximab therapy.

A clinical index of disease activity (Bath Ankylosing Spondylitis Disease Activity Index—BASDAI—range of 0 to 10) [24] was evaluated in all patients at the time of the study. Clinical information on hip involvement, history of synovitis in other peripheral joints and peripheral enthesitis, history of anterior uveitis, presence of syndesmophytes, and HLA-B27 status (typed by cell cytotoxicity) was assessed. Moreover, C-reactive protein (CRP), by a latex immunoturbidity method, erythrocyte sedimentation rate (ESR) (Westergren), serum glucose, total cholesterol, HDL and LDL cholesterol, and triglycerides (fasting overnight determinations) were assessed in all the patients at the time of the study.

The main demographic, clinical, and laboratory data of this series of 30 AS patients at the time of the study are shown in Table 1. Since at that time all patients were undergoing periodical treatment with the anti-TNF-*α* monoclonal antibody infliximab (median duration of periodical treatment with this biologic agent: 23 months), the mean BASDAI ± standard deviation (SD) was only 2.94 ± 2.11.

The local institutional committee approved anti-TNF-*α* therapy. Also, patients gave informed consent to participate in this study. Neither this study nor the former studies on the short-term effect of infliximab therapy on insulin resistance, adipokines, and biomarkers of endothelial cell activation in AS were supported by any pharmaceutical drug company.

### 2.2. Study Protocol

In all cases, the drug was given to patients as an intravenous infusion in a saline solution over 120 minutes. All measurements were made in the fasting state. Blood samples were taken at 0800 hours for determination of the erythrocyte sedimentation rate (ESR) (Westergren), C-reactive protein (CRP) (latex immunoturbidimetry), lipids (enzymatic colorimetry), plasma glucose, and serum insulin (DPC, Dipesa, Los Angeles, CA, USA). As previously described, insulin resistance was estimated by the homeostasis model assessment of insulin resistance (HOMA-IR) using the formula (insulin  (*μ*U/mL) × glucose  (mmol/l) ÷ 22.57) [4]. A commercial ELISA kit was used to measure serum TRAIL levels (R&D Systems, DTRL00); assay  sensitivity = 7.87 pg/mL; intra- and interassay coefficients of variation were <3.9% and <6%, respectively (Minneapolis, MN, USA) according to the manufacturer's instructions. Serum levels of TRAIL were measured in samples obtained immediately prior to an infliximab infusion and 120 minutes later. Total plasma adiponectin and osteoprotegerin levels, serum resistin, leptin, visfatin, apelin, angiopoietin-2 (Angpt-2), asymmetric dimethylarginine (ADMA), gelsolin, osteopontin (OPN), and retinol-binding protein 4 (RBP-4) levels obtained immediately before infliximab infusion were determined by ELISA as previously described [5–12].

### 2.3. Statistical Analyses

Variables were expressed as mean ± SD, median, interquartile range (IQR), or percentages. Correlation between basal TRAIL at time 0 and selected continuous variables was performed, adjusting for age at the time of the study, sex, and classic CV risk factors via estimation of the Pearson partial correlation coefficient (*r*).

The associations between baseline characteristics and serum TRAIL concentrations were assessed by Student's paired *t*-test. Differences in TRAIL levels between men and women and patients with hypertension or without were assessed by the Mann-Whitney *U* test.

TRAIL serum levels before (at time 0) and after infusion (at time 120 minutes) were compared using the paired Student's *t*-test.

Two-sided *P*  values ≤ 0.05 were considered to indicate statistical significance. Analyses were performed using Stata 12/SE (StataCorp, College Station, TX).

## 3. Results

### 3.1. Differences in TRAIL Serum Levels between AS Patients and Controls

**Figure 1:**
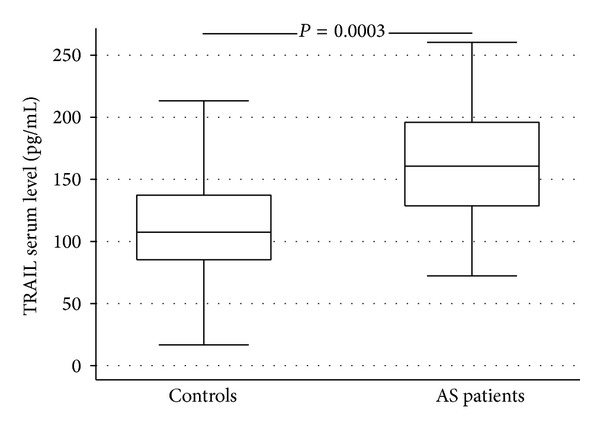
Box plot showing differences between AS patients and healthy controls matched by age, sex, and traditional CV risk factors in TRAIL serum concentration.

TRAIL serum levels were higher in AS patients (mean ± SD: 160.57 ± 47.48 pg/mL; median (IQ range): 160.72 (128.69–195.99)) than in healthy controls (mean ± SD: 117.96 ± 48.20 pg/mL; median (IQ range): 107.49 (85.32–137.31)) (*P* < 0.0003) (Figure 1).

### 3.2. Relationship of TRAIL Concentration with Disease Activity and Clinical Features

Circulating TRAIL levels did not correlate with disease duration, BASDAI, or VAS spinal pain at the time of the study (Table 2). Likewise, no difference in TRAIL concentration was observed when patients with a history of anterior uveitis, presence of syndesmophytes, hip involvement or synovitis in other peripheral joints, and peripheral enthesitis were compared with the remaining patients who did not exhibit these characteristics. It was also the case when patients were compared according to HLA-B27 status (data not shown).

### 3.3. Relationship of Demographic Features, Inflammation, Adiposity, and Adipokines with Circulating TRAIL Levels

TRAIL serum levels did not show significant association with age at the onset of symptoms, BMI, and CRP and ESR at the time of the study and at the time of disease diagnosis (Table 2). Interestingly, we observed an inverse correlation of TRAIL with resistin (*r* = −0.427; *P* = 0.05) and a marginal association with apelin (*r* = 0.373; *P* = 0.06). No association with the rest of adipokines was observed (Table 2). When AS patients were stratified according to sex, no significant differences were found in TRAIL serum levels (data not shown).

### 3.4. Relationship of TRAIL Levels with Metabolic Syndrome Features Other Than Adiposity

TRAIL serum levels displayed an inverse correlation with total cholesterol (*r* = −0.393; *P* = 0.04) and LDL cholesterol (*r* = −0.452; *P* = 0.02). In addition, we observed a negative correlation of TRAIL with insulin sensitivity index (QUICKI: *r* = −0.440; *P* = 0.02) and a marginal association with IR (HOMA-IR: *r* = 0.368; *P* = 0.06) (Table 2). However, we did not observe any association between TRAIL serum levels and systolic or diastolic blood pressure, HDL cholesterol, triglycerides, or serum glucose levels (Table 2). Likewise, we did not find any correlation between TRAIL and RBP-4 levels (Table 2). Besides, no significant differences in TRAIL serum levels were seen when patients were stratified according to the presence or absence of arterial hypertension (not shown).

### 3.5. Relationship of TRAIL Serum Levels with Biomarkers of Endothelial Cell Activation and Atherosclerosis

No correlation of TRAIL levels with Angpt-2 or ADMA was disclosed. However, we found a marginally negative correlation between TRAIL and OPN levels (*r* = −0.372; *P* = 0.06) (Table 2).

### 3.6. Changes in TRAIL Levels upon Infliximab Therapy

TRAIL serum levels did not change following an infliximab infusion. In this regard, the mean ± SD values of TRAIL were 160.57 ± 47.48 pg/mL immediately prior to infliximab infusion (time 0) and 157.06 ± 65.90 pg/mL at the end of the infusion (time 120 minutes) (*P* = 0.772).

## 4. Discussion

TRAIL is a molecule that has been proposed to be involved in the pathophysiology of several autoimmune diseases, not only for its role in the apoptotic process, but also for its functions as an antiatherogenic and anti-inflammatory molecule [25]. Additionally, several pieces of evidence support the potential use of TRAIL as a biomarker of CV disease [14]. Since the implication of TRAIL in AS has not been completely elucidated, in this study we aimed to further clarify the role of this potential biomarker in the pathogenesis and in the mechanisms associated with MeS and CV disease in AS.

In our study, patients with AS displayed low disease activity (mean BASDAI: 2.94) due to prolonged biologic therapy (median duration: 23 months). Interestingly, TRAIL levels were higher in AS patients than in controls. In keeping with our results, increased levels of TRAIL were found in rheumatoid arthritis patients undergoing disease-modifying antirheumatic drug therapy for 1 year when compared to controls [26]. Likewise, lupus patients with inactive disease showed higher TRAIL levels than those with active disease, even higher than those found in controls [25]. Additionally, a study performed on Chinese AS patients also disclosed higher TRAIL levels when compared to healthy controls [27].

Next, we investigated the potential association of TRAIL with metabolic indices, since AS patients often display MeS features [2]. In this regard, we disclosed an inverse correlation of TRAIL with insulin sensitivity index and a marginal association with IR (HOMA-IR). A similar association between TRAIL and HOMA-IR was observed in diabetic patients [28]. It is well known that IR is associated with inflammation [29]; therefore, an increase in the levels of the anti-inflammatory TRAIL molecule would act to compensate such an inflammatory burden.

Furthermore, we also disclosed an inverse correlation of TRAIL with the proinflammatory adipokine resistin and a marginally negative association with the proinflammatory biomarker of endothelial activation OPN. In line with these results, a marginally positive correlation between TRAIL and apelin, an anti-inflammatory adipokine, was also observed. These results further support the previously described anti-inflammatory role of TRAIL [17].

Unlike patients with diabetes or obese individuals [15, 28], in our study that included nondiabetic and mostly nonobese (27 of 30) AS patients, there was a negative correlation between TRAIL levels and total/LDL cholesterol. Chronic inflammation is associated with a paradoxical decrease of lipid levels [30, 31]. Despite having low disease activity probably due to long-term anti-TNF-*α* therapy, a chronic inflammatory state is plausible to exist in our patients (CRP 24 at disease diagnosis and 6.24 mg/L at the time of the study). In this regard, it was reported that CRP values greater than 3 mg/L were associated with high risk of future CV events [32]. Therefore, also in this situation, elevated TRAIL levels may act to compensate the chronic inflammatory state that is present in these patients (reflected by the inverse correlation between TRAIL and total/LDL cholesterol as well as by the presence of CRP levels above the normal range).

Finally, although the aim of the study was to evaluate the immediate direct effect of an infliximab infusion on TRAIL levels, adipokines, biomarkers of endothelial activation, and metabolic syndrome, it may constitute by itself a potential limitation since it is possible that the influence of infliximab on TRAIL levels may require several steps before directly reducing TRAIL levels. Therefore, further studies aimed at determining the effect of infliximab on TRAIL levels at different times, especially at later times, are needed to fully establish the effect of anti-TNF-*α* blockade on TRAIL and other molecules implicated in the mechanisms leading to CV disease in AS.

## 5. Conclusion

In conclusion, our results suggest that elevated TRAIL levels in AS patients may be the result of a compensatory mechanism to reduce CV risk as a result of IR and chronic inflammatory state in these patients.

## Figures and Tables

**Table 1:** Demographic, clinical, and laboratory data of 30 patients with ankylosing spondylitis.

Variable	*n* (%)
Mean age (years) ± SD	
At the time of study	50.47 ± 14.85
At the time of onset of symptoms	28.23 ± 10.40
Delay to the diagnosis (years) ± SD	11.48 ± 9.01
Men/women	21 (70)/9 (30)
Mean disease duration (years) ± SD*	21.97 ± 13.16
History of classic cardiovascular risk factors	
Hypertension (*n* = 30)	12 (40)
Dyslipidemia (*n* = 30)	11 (36.67)
Obesity (BMI > 30 kg/m^2^) (*n* = 30)	3 (10.00)
Current smokers (*n* = 30)	13 (43.33)
Mean blood pressure (mm Hg) ± SD*	
Systolic	123.17 ± 18.17
Diastolic	75.67 ± 12.51
Mean body mass index (kg/m^2^) ± SD	26.70 ± 3.26
Mean BASDAI ± SD*	2.94 ± 2.11
Mean VAS ± SD*	31.13 ± 24.23
Hip involvement, *n* (%) (*n* = 30)	6 (20)
Synovitis and/or enthesitis in other peripheral joints, *n* (%) (*n* = 27)	11 (36.67)
Anterior uveitis, *n* (%) (*n* = 30)	6 (20.00)
Syndesmophytes, *n* (%) (*n* = 30)	10 (33.33)
Mean CRP (mg/L) ± SD**	
At the time of disease diagnosis	24.01 ± 33.43
At the time of study	6.24 ± 8.65
Mean ESR (mm/1st hour) ± SD***	
At the time of disease diagnosis	30.10 ± 28.23
At the time of study	19.00 ± 15.18
Mean cholesterol or triglycerides (mg/dL) ± SD*	
Total cholesterol	199.10 ± 30.61
HDL cholesterol	53.17 ± 12.81
LDL cholesterol	126.77 ± 26.54
Triglycerides	93.97 ± 56.70
Mean fasting serum glucose (mg/dL) ± SD*	92.77 ± 8.63
HLA-B27 positive (*n* = 27)	20 (74.07)

*At the time of the study. **Normal value <5 mg/L. ***Normal value <20 mm/1st hour.

BASDAI: Bath Ankylosing Spondylitis Disease Activity Index; BMI: body mass index; CRP: C-reactive protein; ESR: erythrocyte sedimentation rate; HDL: high-density lipoprotein; HLA: human leukocyte antigen; LDL: low-density lipoprotein; SD: standard deviation; VAS: visual analogue scale.

**Table 2:** Partial correlation of serum TRAIL prior to infliximab infusion (at time 0) with selected continuous variables adjusted for age at the time of the study, sex, and classic cardiovascular risk factors (dyslipidemia, smoking, obesity, and hypertension) in 30 patients with ankylosing spondylitis.

Variable	TRAIL (time 0)	
	*r*	*P*
Age at the onset of symptoms	−0.026	0.90
Disease duration*	−0.011	0.96
BMI*	0.042	0.84
Systolic blood pressure*	0.039	0.85
Diastolic blood pressure*	0.087	0.66
BASDAI*	0.089	0.66
VAS spinal pain*	0.135	0.50
ESR* (natural-log-transformed)	0.018	0.93
CRP* (natural-log-transformed)	−0.080	0.69
ESR** (natural-log-transformed)	0.045	0.83
CRP** (natural-log-transformed)	0.011	0.96
**T** **o** **t** **a** **l** **c** **h** **o** **l** **e** **s** **t** **e** **r** **o** l* (natural-log-transformed)	−**0.393**	**0.04**
HDL cholesterol* (natural-log-transformed)	−0.111	0.58
**L** **D** **L** **c** **h** **o** **l** **e** **s** **t** **e** **r** **o** l* (natural-log-transformed)	−**0.452**	**0.02**
Atherogenic index* (total cholesterol/HDL)	−0.180	0.38
Triglycerides* (natural-log-transformed)	0.185	0.36
Serum glucose* (natural-log-transformed)	−0.092	0.65
**HOMA-IR at time**0*	**0.368**	**0.06**
**QUICKI at time**0*	−**0.440**	**0.02**
**Resistin at time 0**	−**0.427**	**0.05**
Adiponectin at time 0	−0.009	0.97
Leptin at time 0	0.069	0.74
Visfatin at time 0	0.048	0.81
Angpt-2 at time 0	−0.055	0.79
**Apelin at time 0**	**0.373**	**0.06**
ADMA at time 0	−0.058	0.78
Gelsolin at time 0	0.173	0.39
**OPN at time 0**	−**0.372**	**0.06**
RBP-4 at time 0	0.318	0.11
Osteoprotegerin at time 0	0.044	0.83

*At the time of the study. **At the time of disease diagnosis.

ADMA: asymmetric dimethylarginine; Angpt-2: angiopoietin 2; BASDAI: Bath Ankylosing Spondylitis Disease Activity Index; BMI: body mass index; CRP: C-reactive protein; ESR: erythrocyte sedimentation rate; HDL: high-density lipoprotein; HOMA-IR: homeostasis model assessment of insulin resistance; LDL: low-density lipoprotein; OPN: osteopontin; QUICKI: quantitative insulin sensitivity check index; RBP-4: retinol-binding protein 4; VAS: visual analogue scale.

## References

[1] González-Juanatey C, Vázquez-Rodríguez TR, Miranda-Filloy JA (2009). The high prevalence of subclinical atherosclerosis in patients with ankylosing spondylitis without clinically evident cardiovascular disease. *Medicine*.

[2] Mathieu S, Motreff P, Soubrier M (2010). Spondyloarthropathies: an independent cardiovascular risk factor?. *Joint Bone Spine*.

[3] Heldmann F, Brandt J, van der Horst-Bruinsma IE (2011). The European ankylosing spondylitis infliximab cohort (EASIC): a European multicentre study of long term outcomes in patients with ankylosing spondylitis treated with infliximab. *Clinical and Experimental Rheumatology*.

[4] Miranda-Filloy JA, Llorca J, Carnero-López B, González-Juanatey C, Blanco R, González-Gay MA (2012). TNF-*α* antagonist therapy improves insulin sensitivity in non-diabetic ankylosing spondylitis patients. *Clinical and Experimental Rheumatology*.

[5] Genre F, Miranda-Filloy JA, López-Mejías R (2013). Antitumour necrosis factor-*α* therapy modulates angiopoietin-2 serum levels in non-diabetic ankylosing spondylitis patients. *Annals of the Rheumatic Diseases*.

[6] Genre F, López-Mejías R, Miranda-Filloy JA (2014). Correlation between two biomarkers of atherosclerosis, osteopontin and angiopoyetin-2, in non-diabetic ankylosing spondylitis patients undergoing TNF-*α* antagonist therapy. *Clinical and Experimental Rheumatology*.

[7] Miranda-Filloy JA, López-Mejías R, Genre F (2013). Adiponectin and resistin serum levels in non-diabetic ankylosing spondylitis patients undergoing TNF-*α* antagonist therapy. *Clinical and Experimental Rheumatology*.

[8] Genre F, López-Mejías R, Miranda-Filloy JA (2014). Antitumour necrosis factor-*α* treatment reduces retinol-binding protein 4 serum levels in non-diabetic ankylosing spondylitis patients. *Annals of the Rheumatic Diseases*.

[9] Genre F, Miranda-Filloy JA, López-Mejías R (2013). Apelin serum levels in non-diabetic ankylosing spondylitis patients undergoing TNF-*α* antagonist therapy. *Clinical and Experimental Rheumatology*.

[10] Genre F, López-Mejías R, Miranda-Filloy JA (2013). Asymmetric dimethylarginine serum levels in non-diabetic ankylosing spondylitis patients undergoing TNF-*α* antagonist therapy. *Clinical and Experimental Rheumatology*.

[11] Genre F, López-Mejías R, Miranda-Filloy JA (2014). Gelsolin levels are decreased in ankylosing spondylitis patients undergoing anti-TNF-alpha therapy. *Clinical and Experimental Rheumatology*.

[12] Genre F, López-Mejías R, Miranda-Filloy JA Osteoprotegerin correlates with disease activity and endothelial activation in non-diabetic ankylosing spondylitis patients undergoing TNF-alpha antagonist therapy.

[13] van Sijl AM, van Eijk IC, Peters MJ (2013). Tumour necrosis factor blocking agents and progression of subclinical atherosclerosis in patients with ankylosing spondylitis. *Annals of the Rheumatic Diseases*.

[14] Bernardi S, Milani D, Fabris B, Secchiero P, Zauli G (2012). TRAIL as biomarker and potential therapeutic tool for cardiovascular diseases. *Current Drug Targets*.

[15] Brombo G, Volpato S, Secchiero P (2013). Association of soluble Tumor necrosis factor-Related Apoptosis-Inducing Ligand (TRAIL) with central adiposity and low-density lipoprotein cholesterol. *PLoS ONE*.

[16] Secchiero P, Corallini F, Beltrami AP (2010). An imbalanced OPG/TRAIL ratio is associated to severe acute myocardial infarction. *Atherosclerosis*.

[17] Secchiero P, Rimondi E, di Lasio MG (2013). C-reactive protein downregulates TRAIL expression in human peripheral monocytes via an Egr-1-dependent pathway. *Clinical Cancer Research*.

[18] di Bartolo BA, Cartland SP, Harith HH, Bobryshev YV, Schoppet M, Kavurma MM (2013). TRAIL-deficiency accelerates vascular calcification in atherosclerosis via modulation of RANKL. *PLoS ONE*.

[19] van der Linden S, Valkenburg HA, Cats A (1984). Evaluation of diagnostic criteria for ankylosing spondylitis. A proposal for modification of the New York criteria. *Arthritis and Rheumatism*.

[20] Gonzalez-Gay MA, Llorca J, Garcia-Unzueta MT (2008). High-grade inflammation, circulating adiponectin concentrations and cardiovascular risk factors in severe rheumatoid arthritis. *Clinical and Experimental Rheumatology*.

[21] González-Gay MA, García-Unzueta MT, González-Juanatey C (2008). Anti-TNF-alpha therapy modulates resistin in patients with rheumatoid arthritis. *Clinical and Experimental Rheumatology*.

[22] Gonzales-Gay MA, Garcia-Unzueta MT, de Matias JM (2006). Influence of anti-TNF-*α* infliximab therapy on adhesion molecules associated with atherogenesis in patients with rheumatoid arthritis. *Clinical and Experimental Rheumatology*.

[23] van der Heijde D, Sieper J, Maksymowych WP (2011). 2010 update of the international ASAS recommendations for the use of anti-TNF agents in patients with axial spondyloarthritis. *Annals of the Rheumatic Diseases*.

[24] Garrett S, Jenkinson T, Kennedy LG, Whitelock H, Gaisford P, Calin A (1994). A new approach to defining disease status in ankylosing spondylitis: the bath ankylosing spondylitis disease activity index. *Journal of Rheumatology*.

[25] Lub-De Hooge MN, de Vries EGE, de Jong S, Bijl M (2005). Soluble TRAIL concentrations are raised in patients with systemic lupus erythematosus. *Annals of the Rheumatic Diseases*.

[26] Secchiero P, Corallini F, Castellino G (2010). Baseline serum concentrations of TRAIL in early rheumatoid arthritis: relationship with response to disease-modifying antirheumatic drugs. *Journal of Rheumatology*.

[27] Yang Z-X, Yan L, Hao W, Ye Z, Chang L, Zhong R-Q (2008). Preliminary clinical measurement of the expression of TNF-related apoptosis inducing ligand in patients with ankylosing spondylitis. *Journal of Clinical Laboratory Analysis*.

[28] Kawano N, Mori K, Emoto M (2011). Association of serum TRAIL levels with atherosclerosis in patients with type 2 diabetes mellitus. *Diabetes Research and Clinical Practice*.

[29] Shoelson SE, Lee J, Goldfine AB (2006). Inflammation and insulin resistance. *Journal of Clinical Investigation*.

[30] Robertson J, Peters MJ, McInnes IB, Sattar N (2013). Changes in lipid levels with inflammation and therapy in RA: a maturing paradigm. *Nature Reviews: Rheumatology*.

[31] González-Gay MA, González-Juanatey C Inflammation and lipid profile in rheumatoid arthritis: bridging an apparent paradox.

[32] Ridker PM, Cook N (2004). Clinical usefulness of very high and very low levels of C-reactive protein across the full range of framingham risk scores. *Circulation*.

